# Scrotal calcinosis due to resorption of cyst walls: a case report

**DOI:** 10.1186/1752-1947-2-375

**Published:** 2008-12-08

**Authors:** Alper Parlakgumus, Emine T Canpolat, Kenan Calıskan, Tamer Colakoglu, Sedat Yıldırım, Ali Ezer, Turgut Noyan

**Affiliations:** 1 Department of General Surgery, Faculty of Medicine, Baskent University, Ankara, Turkey; 2 Department of Pathology, Faculty of Medicine, Baskent University, Ankara, Turkey

## Abstract

**Introduction:**

Scrotal calcinosis is a rare benign entity defined as the presence of multiple calcified nodules within the scrotal skin. There are controversies about the origin of this entity. In fact, it is still debatable whether scrotal calcinosis is an idiopathic growth or dystrophic calcification of dartoic muscles. It is also unclear whether scrotal calcinosis originates from inflammation of epidermal cysts affected by mild to moderate inflammation of mononuclear cells, from foreign body granuloma formation followed by resorption of cyst walls or from eccrine epithelial cysts.

**Case presentation:**

We report a 41-year-old male Turkish patient presenting with a 10-year history of scrotal tumours increasing slowly in size and number. Histopathologically, there was no epithelial lining around the calcified nodules, but there was fibrosis adjacent to atrophic stratified squamous epithelium.

**Conclusion:**

Results of histopathological examinations suggested that scrotal calcinosis might have been due to resorption of cyst walls. Surgery remains the key for this problem. In cases of non-massive scrotal calcinosis, like the case presented here, excision of the nodules from the affected part of the scrotal wall and repairing the defect with horizontal stitches offer good cosmetic results without relapse.

## Introduction

Scrotal calcinosis (SC) is a rare benign entity characterized by calcium deposits within the dermis of the scrotal skin. The nodules may range from one to a hundred in number and from 1 mm to several centimetres in size. These nodules are confined to the scrotum and are mostly asymptomatic. SC may not cause any abnormalities in parathyroid hormone, calcitonin, 25-OH vitamin D and phosphor/calcium levels. Few cases of scrotal calcinosis have been reported in the literature and there is still a controversy about the pathogenesis of this rare condition. It is not known whether it is idiopathic or not [1]. Despite the confusion with the origin of this condition, surgery seems to be the treatment of choice for resolving the problem.

## Case presentation

**Figure 1:**
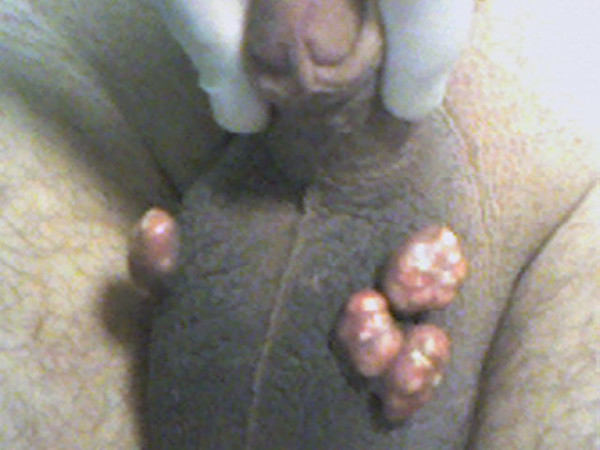
Four buds of nodular lesions on scrotum.

A 41-year-old male Turkish patient presented with a 10-year history of four buds of expansive nodular lesions and a feeling of heaviness in the scrotum. His medical history revealed no remarkable feature of a metabolic disease or hormonal derangement, trauma, sexually transmitted infection or drug abuse. Cutaneous examination showed multiple, hard, palpable, firm nodules within the scrotal skin (Figure 1). Complete blood count and calcium, phosphorus, parathyroid hormone, calcitonin and 25-OH vitamin D levels were normal. The lesions were locally excised from the affected part of the scrotal wall and the patient was well after 2-year follow-up and no recurrence was seen during the follow-up period.

**Figure 2:**
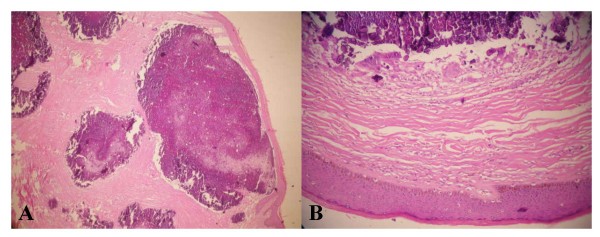
(a) Calcified intradermal nodule lacking an epithelial lining. (b) Original magnification of the same nodule composed of basophilic calcified material without epithelial lining showing amorphous form. Foreign giant cells and fibrosis are present adjacent to atrophic stratified squamous epithelium (haematoxylin and eosin staining ×200).

Excised specimens were 1.5 to 2.5 cm in size. On histological examination, the nodules were composed of basophilic calcified material and located in the dermis while foreign giant cells and fibrosis were present adjacent to the atrophic stratified squamous epithelium. There was no epithelial lining around the calcified nodules (Figure 2a, b).

Immunohistochemically, carcinoembryonic antigen (CEA) was negative while cytokeratin was positive on the surface of the epithelial lining of the scrotum.

## Discussion

Scrotal calcinosis, first described by Lewinski, usually appears in men aged 20 to 40 years. The youngest and oldest patients reported in the literature were 9 and 85 years old, respectively [2].

Scrotal calcinosis consists of nodules within the dermis of the scrotal skin varying in size and number and develops slowly over many years. Although they are mostly asymptomatic, with a feeling of heaviness in the scrotum, discharge and itching are the most frequently encountered complaints [3]. For diagnosis of scrotal calcinosis, Ito *et al. *performed an immunohistochemical study using antibodies against CEA, epithelial membrane antigen (EMA), and gross cystic disease fluid protein-15 (GCDFP-15) to describe dystrophic scrotal calcinosis originating from eccrine cysts. They found a positive reaction for CEA and EMA in the luminal cells and in the contents of a large cyst and ductal structures, and positive GCDFP-15 staining in the latter [4]. Dini and Colafranceschi used antibodies against low molecular weight cytokeratin CAM 5.2, a cocktail of cytokeratin AE1/AE3, CEA, collagen type IV and laminin of the basement membrane and only observed a slight positivity for cytokeratin AE1/AE3 within the amorphous calcified mass, which was probably due to dystrophic calcification of epidermoid cysts [5]. A firm diagnosis can only be made with histological examination as in the case presented here.

In the literature, scrotal calcinosis has rarely been reported and there is an ongoing debate about the pathogenesis of this rare condition. In fact, it is still arguable whether it is idiopathic or not [1]. In their series of 14 cases, Shapiro *et al. *reported that calcified nodules without epithelial linings were idiopathic [6]. However, King *et al. *claimed that the lesions showed dystrophic calcifications of the dartoic muscle [7]. Dini and Colafranceschi noted that inflammation and rupture of epidermoid cysts constituted the main pathological mechanism in SC [5]. Two studies suggested that the epithelial lining may be obscured in the course of time by inflammation of epidermal cysts followed by calcification, rupture of the cyst wall and granulomatous proliferation [8,9]. Swinehart and Golitz thought that scrotal calcinosis resulted from inflammation and calcification.

Furthermore, Song *et al. *examined 51 nodules excised from a patient with SC [9]. They demonstrated that epidermal cysts were affected by mild to moderate inflammation and that mononuclear cell or foreign body granuloma formation was followed by resorption of cyst walls and keratinous material until the calcified deposits remained. One of the most important observations was the resorption of the cyst wall and this was a rapid stage of the sequence. As a result, histopathological findings change depending on the age of cysts and this causes long-term cysts to have fewer or no epithelial lining cells.

The last theory, proposed by Ito *et al. *is that that SCs originate from eccrine epithelial cysts. Matrix debris is deposited following discharge. Antibodies against sulphated mucopolysaccharides and immunohistochemical studies using CEA and EMA give positive reactions [4].

In our patient, no evidence of cystic structure was found around the calcified material and CEA was negative. This suggested that SCs did not result from eccrine epithelial cysts as proposed by Ito *et al. *Although there was no epithelial lining around the calcified nodule, there was fibrosis adjacent to the atrophic stratified squamous epithelium. This type of histopathology was consistent with the findings of Song *et al.*

Scrotal calcinosis can be confused with other lesions. Testicular tumours such as teratomas, gonadoblastomas, and Leydig cell tumours may show calcification or ossification [2]. Scrotal calculi are also found in a secondary hydrocele, thus rendering them impalpable. During ultrasonographic (US) examination, calcification in or adjacent to epididymis may be found and this is usually due to chronic epididymitis. Granulomatous disease should always be considered in these circumstances. Haematoma and sperm granulomas (sperm extravasation with granuloma formation) may produce a solitary echogenic area within the epididymis. The appendix epididymis and appendix testis may calcify and these are recognized by their characteristic position and shape. These lesions are related to previous inflammatory diseases of the epididymis [10].

Subtotal excision of the scrotal wall is recommended for the treatment of massive calcinosis [11]. In the case presented here, there was not a massive occurrence and the lesions were locally excised from the affected part of the scrotal wall. We repaired the defects with horizontal mattress sutures and the stitches were spaced as far as possible from each other on the affected side in order to facilitate good healing and avoid scarring. At the end of a 2-year follow-up, we achieved satisfactory cosmetic results and there was no relapse. However, there have been some patients who have developed recurrent nodules in the scrotal corium after primary excision. Recurrent asymptomatic lesions proving to be calcinosis by biopsy may be observed safely because of carrying no risk of malignancy. Therefore, recurrent asymptomatic lesions may be followed up due to their clinical circumstance [2].

In fact, SC is of interest to multiple disciplines such as urology, plastic and general surgery and pathology. Because most patients with scrotal calcinosis are asymptomatic, they usually seek medical advice for cosmetic reasons. Therefore, biopsy of these lesions at an advanced stage can be late and only shows dermal calcium deposits [2]. In such cases, the patients should be assured that they do not necessarily have a malignant condition, but that only a histological examination of the material removed through surgery can confirm it. They should also be reminded that the disease may recur, though rarely, and that the most appropriate treatment is surgery.

## Conclusion

SC is of interest to multiple disciplines such as urology, plastic and general surgery, and pathology. Although the pathogenesis and basic origin of scrotal calcinosis are controversial, surgical excision seems to be the gold standard for treatment of the condition, and the surgical approach should be based on the extent of the nodules.

## Consent

Written informed consent was obtained from the patient for publication of this case report and any accompanying images. A copy of the written consent is available for review by the Editor-in-Chief of this journal.

## Competing interests

The authors declare that they have no competing interests.

## Authors' contributions

AP analysed and interpreted the patient data, ETC performed the histological examination of scrotal calcinosis while KC assisted with the interpretation. TC was involved in the surgical approach. Contributions have made substantive intellectual contributions to this study. SY, AE, TN had also made substantive intellectual contributions of the article; SY and TN have made substantial contributions to conception and design of the manuscript, revised it critically for intellectual content and gave final approval of the version to be published. AE both took part in aquisition of data and drafting of the manuscript.
